# The Treatment of Cardiac Failure in Chronic Lung Affections

**Published:** 1905-11-18

**Authors:** 


### THE TREATMENT OF CARDIAC FAILURE IN CHRONIC LUNG AFFECTIONS.

Professor Julius Dreschfeld,[1] speaking of the effects of chronic bronchitis and emphysema on the heart, emphasises the importance of noting any signs of cardiac failure in these conditions. To meet such failure he outlines the following scheme of treatment: Digitalis, in the form either of digitalin or of the dried leaves, is most successful when there is no considerable œdema or ascites. When these latter symptoms are pronounced calomel, or magnesium sulphate, or compound jalap powder is indicated. To increase diuresis, and especially when the urine is scanty and there is albuminuria either from passive congestion of the kidney or from Bright's disease, the acetate of sodium theocin is useful. In sleeplessness and cardiac dyspnœa small doses of morphine with atropine may be given. Arterio-sclerosis demands administration of one or other of the nitrites. For great enlargement of the liver repeated doses of calomel and the free application of leeches to the hypochondrium are suggested. In advanced cases rest is a necessity, but in others gentle and graduated exercises are indicated. Carbonic acid baths, such as those of Nauheim, are of value, and with marked dilatation of the right heart and distension of the veins venesection should be practised. Withdrawal of fluid from the serous cavities and from the subcutaneous tissues of the lower limbs is also often useful. In dieting a patient with cardiac failure care should be taken not to overload the stomach; meals should be small and taken at frequent intervals; fluids should be given in limited quantities; and alcohol should be used as a medicine only and not as an article of diet.

[1] Brit. Med. Jour., October 21, 1905.

